# Long non-coding RNA CCHE1 participates in postoperative distant recurrence but not local recurrence of osteosarcoma possibly by interacting with ROCK1

**DOI:** 10.1186/s12891-020-3184-x

**Published:** 2020-07-13

**Authors:** Zhi Zhang, Tao Yu, Wei Geng

**Affiliations:** Department of Orthopedics, Liaocheng People’s Hospital, No. 67 Dongchang West Road, Dongchangfu District, Liaocheng City, Shandong Province 252000 People’s Republic of China

**Keywords:** Osteosarcoma, Follow-up, Distant recurrence, Local recurrence

## Abstract

**Background:**

Clinical treatment of osteosarcoma suffers from high recurrence rate. Therefore, is of great clinical values to develop predictive markers for recurrent osteosarcoma. Cervical carcinoma high-expressed lncRNA 1 (lncRNA CCHE1) participates in several types of malignancies, while its functionality in osteosarcoma is unknown. This study was therefore carried out to explore the involvement of lncRNA CCHE1 in recurrent osteosarcoma.

**Methods:**

A total of 87 osteosarcoma patients received surgical resection and 38 healthy volunteers were included in this study. The 87 osteosarcoma patients were followed up for 5 years to record the recurrence of osteosarcoma. Plasma levels of lncRNA CCHE1 and ROCK1 on the day of discharge and during follow-up were measured by real-time quantitative PCR and ELISA, respectively. The effects of CCHE1 siRNA silencing on ROCK1 expression were analyzed by real-time quantitative PCR and western blot. Transwell assay was performed to analyze the role of lncRNA CCHE1 and ROCK1 in regulating cell invasion and migration.

**Results:**

We observed that, on the day of discharge, plasma lncRNA CCHE1 was upregulated in osteosarcoma patients who developed distant recurrence (DR) during follow-up than in osteosarcoma patients who developed local recurrence (LR), patients with non-recurrence (NR) and healthy controls. On the day of discharge, plasma levels of ROCK1 were higher in DR, LR and NR groups in comparison to healthy controls. On the day of discharge, plasma levels of lncRNA CCHE1 were positively correlated with plasma levels of ROCK1 only in patients who developed DR during follow-up, but not in patients who developed LR, NR and control groups. During follow-up, plasma levels of lncRNA CCHE1 were further increased in DR group but slightly decreased in LR and NR groups. LncRNA CCHE1 siRNA silencing inhibited, while ROCK1 overexpression promoted osteosarcoma cell invasion and migration. ROCK1 overexpression attenuated the role of CCHE1. LncRNA CCHE1 siRNA silencing led to inhibited ROCK1 expression in cancer cells.

**Conclusion:**

Therefore, lncRNA CCHE1 may participate in postoperative distant recurrence of osteosarcoma caner possibly by interacting with ROCK1 to promote cancer cell invasion and migration.

## Background

With the development of early diagnosis programs and surgical resection techniques, treatment outcomes of cancer at early stages have been significantly improved [1, 2]. However, postoperative survival of cancer patients is still challenged by the higher recurrence rate, which is a major cause of poor survival [3, 4]. As the most common histological type of primary bone cancer, osteosarcoma is a rare malignancy that only affect less than 1 per 100,000 [5]. However, increasing incidence rate of this disease was observed during the past several decades [6]. Although surgical resection can reach radical treatment in many cases of osteosarcoma patients, recurrence inevitably occur in a considerable portion of osteosarcoma patients, lead to poor survival [6]. Therefore, how to further improve the treatment and prevent postoperative recurrence of osteosarcoma is still a focus of the clinical treatment of osteosarcoma.

Rho-associated protein kinase 1 (ROCK1) is a major downstream effector of the small GTPase RhoA that plays pivotal roles in the regulation of cell motility [7]. In cancer biology, ROCK1 promotes cancer development and progression by mediating tumor metastasis [8]. At present, inhibition of ROCK1 has shown promising application values in the treatment of different types of malignancies [9, 10]. However, studies on the functions and clinical applications of ROCK1 in osteosarcoma are rare. LncRNA CCHE1 has been reported to be an oncogenic lncRNA that participates in several types of malignancies [11–14], while its involvement in osteosarcoma is unknown. Therefore, it will be interesting to investigate the roles of lncRNA CCHE1 in osteosarcoma and to explore its interactions with ROCK1. In the present study we showed that lncRNA CCHE1 could participate in postoperative distant recurrence of osteosarcoma possibly by interacting with ROCK1.

## Methods

### Cell lines and human plasma samples

Two osteosarcoma cell lines MG-63 and U2OS from ATCC (USA) were used. EMEM medium supplemented with 10% FBS was used to cultivate cancer cells under normal conditions (37 °C, 5% CO_2_).

Plasma samples were derived from 87 osteosarcoma patients and 38 healthy volunteers, who were admitted by Liaocheng People’s Hospital from January 2011 to May 2013. Patients’ inclusion criteria: 1) patients diagnosed as osteosarcoma at stage I and II for the first time by pathological examinations; 2) patients signed informed consent. Patients’ exclusion criteria: 1) patients with initiated therapy; 2) patients who were suffering from multiple diseases. All patients received surgical resection. Ethics committee of Liaocheng People’s Hospital approved this study.

### Follow-up and grouping

A 5-year follow-up was performed after discharge. Distant recurrence (DR) occurred in 18 cases, local recurrence (LR) in 21 cases and non-recurrence (NR) in 48 cases.

### Blood extractions on the day of discharge and during follow-up

Blood was extracted on the day of discharge, on the day of the diagnosis of DR or LR during follow-up or on the last day of follow-up in cases of NR. See Table 1 for patients’ information.

**Table 1:** Clinical data of 4 groups of participants

	DR	LR	NR	Control
Cases	18	21	48	38
Mean age (years)	46.1 ± 4.2	45.4 ± 3.9	46.3 ± 5.1	44.6 ± 3.8
Gender				
Male	10	11	26	19
Female	8	10	22	19
AJCC stage				
I	6	8	18	
II	12	13	30	

### Real-time quantitative PCR

Monarch® Total RNA Miniprep Kit (NEB) was used to isolate RNA. RT-qPCR was performed using BlazeTaq™ One-Step SYBR Green RT-qPCR Kit (Genecopoeia). Primers used in PCR reactions were: 5′-AAGGTCCCAGGATACTCGC-3′ (forward) and 5′-GTGTCGTGGACTGGCAAAAT-3′ (reverse) for lncRNA CCHE1; 5′-ACCTGTAACCCAAGGAGATGTG-3′ (forward) and 5′-CACAATTGGCAGGAAAGTGG-3′ (reverse) for ROCK1; 5′-CAGGAGGCATTGCTGATGAT-3′ (forward) and 5′-GAAGGCTGGGGCTCATTT-3′ (reverse) for GAPDH. Thermal conditions for PCR reactions were: 95 °C for 45 s, then 95 °C for 16 s and 58.5 °C for 42 s for 40 cycles. Ct values were processed using 2^**-**ΔΔCT^ methods.

### ELISA

Plasma levels of ROCK1 were measured using ROCK1 ELISA Kit (Human) (OKEH06554, Aviva Systems Biology). All steps were completed according to manufacturer’s instructions. Plasma levels of ROCK1 were expressed as ng/ml.

### Cell transfection

ROCK1 expression vector, lncRNA CCHE1 siRNA (5′-GCTTCTGACCAGCGACGCTAGGAGTAGCTG-3′) and negative control (NC) siRNA were from GenePharma (Shanghai, China). Cancer cells were cultivated to reach 80–90% confluence and transfection was carried out using lipofectamine 2000 (11668–019, Invitrogen, Carlsbad, USA) reagent with vectors and siRNAs at 10 nM and 50 nM, respectively. All steps were completed according to manufacturer’s instructions. Control group included untransfected cells and NC cells were empty vector- or NC siRNA-transfected cells. Cells were harvested at 24 h after transfection.

### Transwell migration and invasion assay

Overexpression rate of ROCK1 above 200% and knockdown rate of lncRNA CCHE1 below 50% were reached before this experiment. Briefly, 3000 cells in 0.1 ml medium were transferred to the upper chamber and medium containing 20% FBS was used to fill the lower chamber. After 24 h, membranes were cleaned and 0.5% crystal violet (Sigma-Aldrich, USA) staining was performed in dark for 18 min at room temperature. This protocol was used for both migration and invasion assay except that the upper chamber was not pre-coated before invasion assay. Similar results, in terms of cell migration and invasion were obtained from two negative controls. Therefore, only one negative control is shown in results section for simplification.

### Western-blot

Knockdown rate of lncRNA CCHE1 were reached before this experiment. RIPA (Bio-Rad) was used to isolate proteins. Proteins were separated using 12% SDS-PAGE gel. Western blotting was performed using conventional method. Primary antibodies included rabbit anti-human ROCK1 (1: 1200, ab97592, Abcam) and rabbit anti-human GAPDH antibody (1: 1200, ab37168, Abcam). IgG-HRP (1:1000, MBS435036, MyBioSource) was used as the secondary antibody. ECL (Sigma-Aldrich, USA) was used for signal development and data normalization was performed using Image J software.

### Statistical analysis

Mean ± standard deviation values were used to express the data of 3 replicates. Comparisons of plasma levels of CCHE1 and ROCK1 among different groups as well as cell migration and invasion and ROCK1 expression among cells with different treatments were performed using one-way ANOVA and Tukey test. Comparisons of plasma levels of CCHE1 measure on the discharge and during follow-up levels were performed by paired test. Correlation analyses were performed using Pearson correlation coefficient. *p* < 0.05 was statistically significant.

## Results

### Differential expression of plasma lncRNA CCHE1 in different groups

**Fig. 1:**
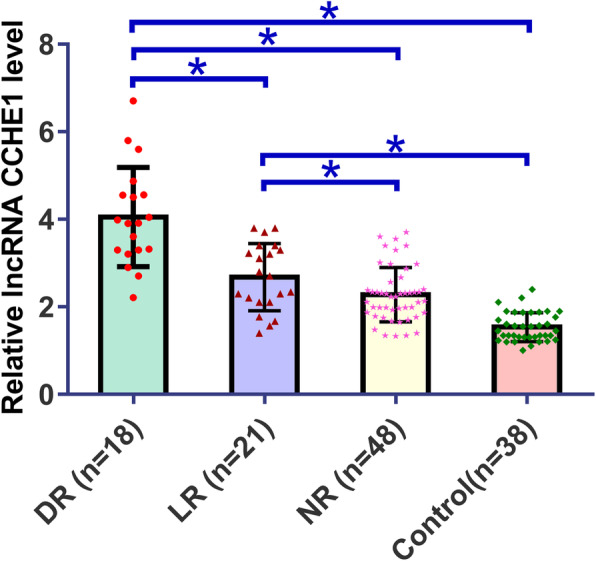
Differential expression of lncRNA CCHE1 in different groups (*, *p* < 0.05)

QRT-PCR results revealed that, on the day of discharge, plasma lncRNA CCHE1 was upregulated in osteosarcoma patients who developed DR during follow-up than in osteosarcoma patients who developed LR, patients with NR and controls (Fig. 1, *p* < 0.05). Levels of plasma lncRNA CCHE1 were also higher in osteosarcoma patients with LR and patients with NR than in healthy controls (Fig. 1, *p* < 0.05). No significant differences in plasma levels of lncRNA CCHE1 were found between LR and NR groups (Fig. 1, *p* > 0.05).

### Differential expression of plasma ROCK1 in different groups

**Fig. 2:**
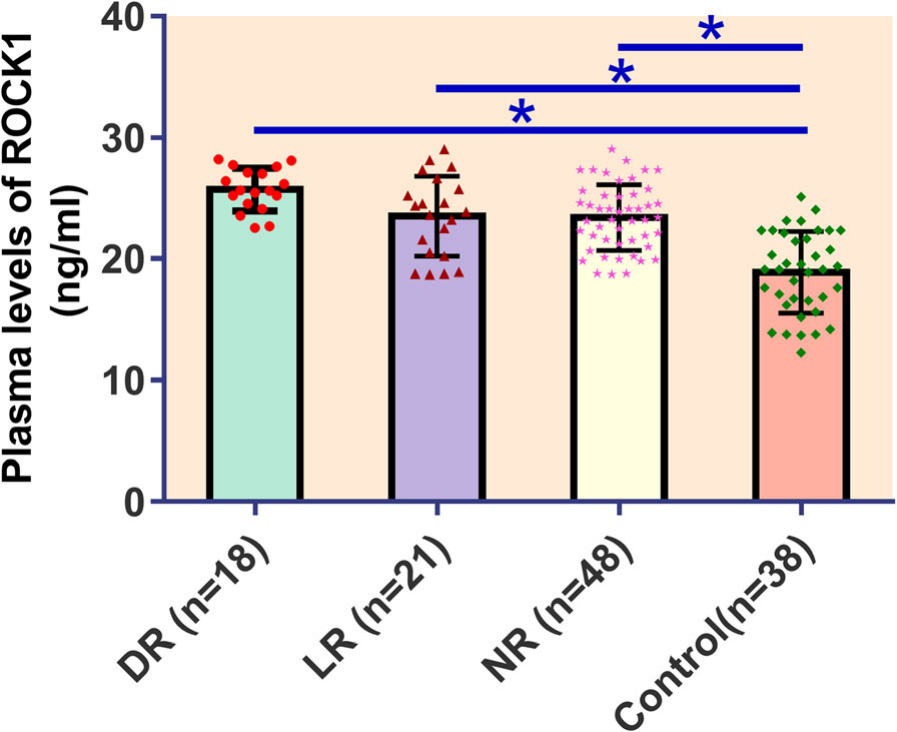
Differential expression of plasma ROCK1 in different groups (*, p < 0.05)

ELISA results revealed that, on the day of discharge, plasma ROCK1 was upregulated in DR, LR and NR groups than in controls (Fig. 2, *p* < 0.05). No significant differences in plasma levels of ROCK1 were found among DR, LR and NR groups (Fig. 2, *p* > 0.05).

### Plasma levels of lncRNA CCHE1 were positively correlated with plasma levels of ROCK1 only in DR group

**Fig. 3:**
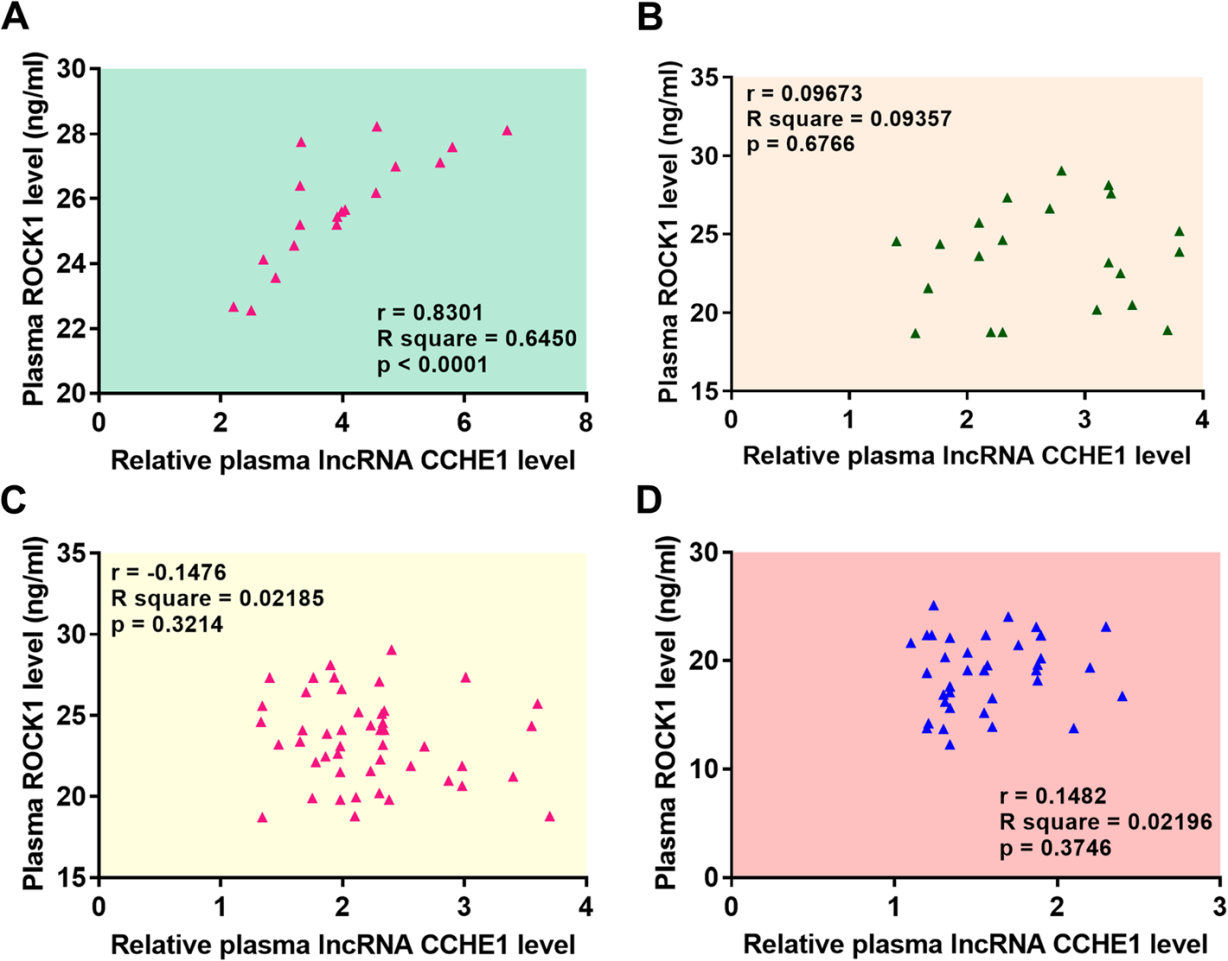
Plasma levels of lncRNA CCHE1 were positively correlated with plasma levels of ROCK1 only in DR group. Plasma levels of lncRNA CCHE1 were positively correlated with plasma levels of ROCK1 in DR group (**a**), but not in LR (**b**), NR (**c**) and control group (**d**)

On the day of discharge, Pearson correlation coefficient analysis showed that plasma levels of lncRNA CCHE1 were positively correlated with plasma levels of ROCK1 in DR group (Fig. 3a). In contrast, the correlation between plasma levels of ROCK1 and lncRNA CCHE1 were not significant in LR (Fig. 3b), NR (Fig. 3c) and control group (Fig. 3d).

### Plasma levels of lncRNA CCHE1 were further increased during follow-up only in DR group

**Fig. 4:**
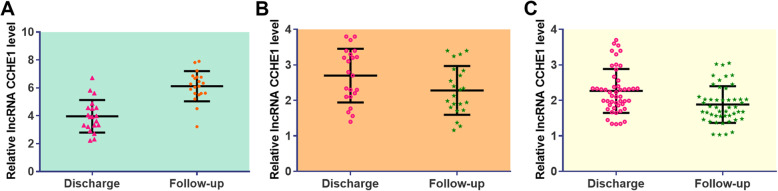
Plasma levels of lncRNA CCHE1 were further increased during follow-up only in DR group. Compared with plasma levels of lncRNA CCHE1 on the day of discharge, significantly upregulated plasma lncRNA CCHE1 during follow-up was observed in DR group (**a**) but not in LR (**b**) and NR (**c**) groups (*, *p* < 0.05)

Compared with plasma levels of lncRNA CCHE1 on the day of discharge, significantly upregulated plasma lncRNA CCHE1 during follow-up was observed in DR group (Fig. 4a, *p* < 0.05). In contrast, slightly decreased plasma levels of lncRNA CCHE1 were observed in LR (Fig. 4b) and NR (Fig. 4c) groups.

### LncRNA CCHE1 silencing inhibited migration and invasion of cells of MG-63 and U2OS human osteosarcoma cell lines

**Fig. 5:**
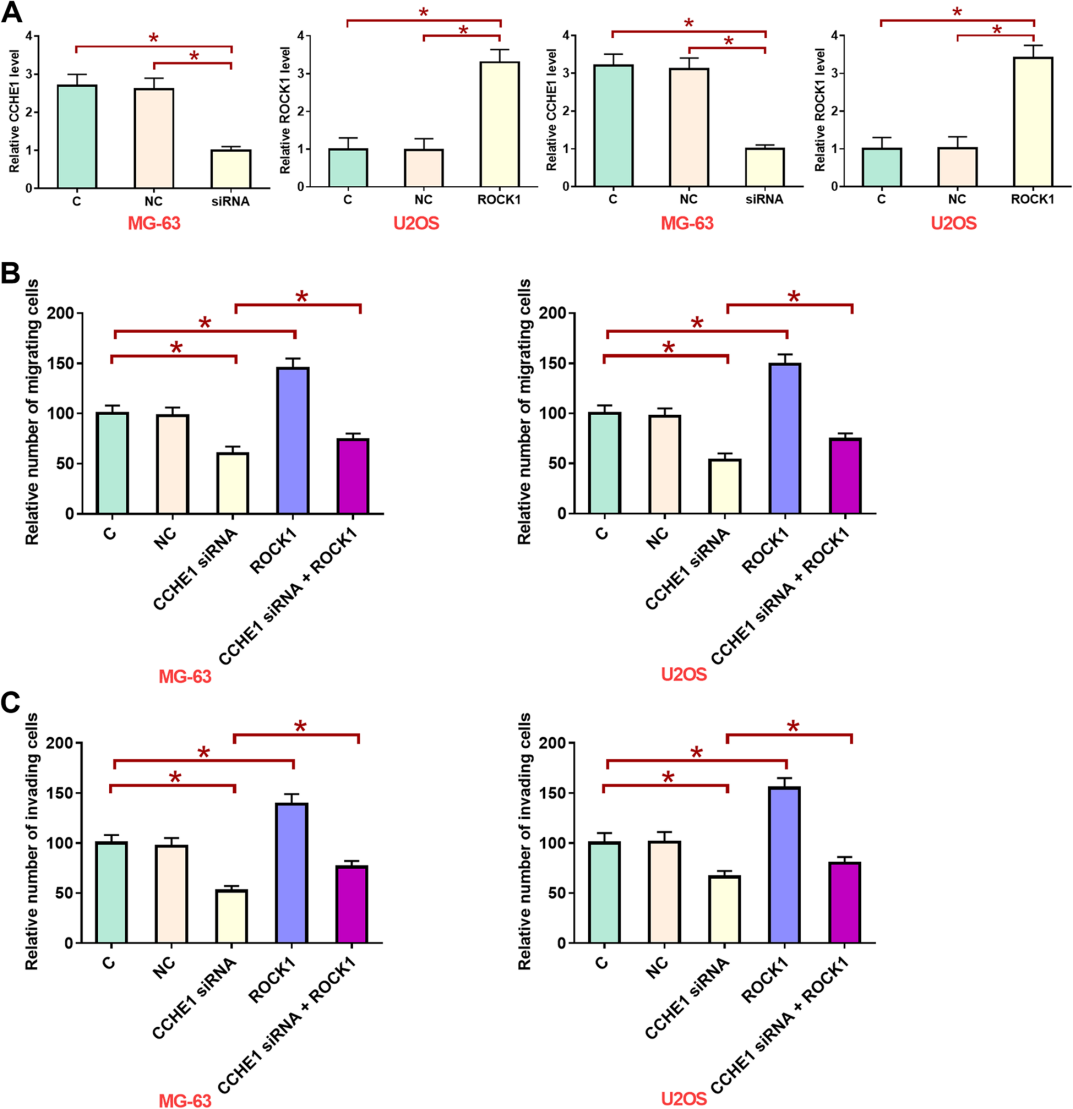
LncRNA CCHE1 silencing inhibited migration and invasion of cells of MG-63 and U2OS human osteosarcoma cell lines. LcRNA CCHE1 silencing and OCK1 overexpression were reached at 24 h after transfection (**a**). LncRNA CCHE1 silencing significant inhibited, while ROCK1 overexpression significantly promoted the migration (**b**) and invasion (**c**) of cells of both MG-63 and U2OS human osteosarcoma cell lines. In addition, ROCK1 overexpression attenuated the inhibitory effects of lncRNA CCHE1 on cancer cell migration and invasion (*, *p* < 0.05)

LcRNA CCHE1 silencing and OCK1 overexpression were reached at 24 h after transfection (Fig. 5a, *p* < 0.05). Compared with control group (C) and negative control group (NC), lncRNA CCHE1 silencing significant inhibited, while ROCK1 overexpression significantly promoted the migration (Fig. 5b) and invasion (Fig. 5c) of cells of both MG-63 and U2OS human osteosarcoma cell lines (*p* < 0.05). In addition, ROCK1 overexpression attenuated the inhibitory effects of lncRNA CCHE1 on cancer cell migration (Fig. 5b) and invasion (Fig. 5c) (*p* < 0.05).

### LncRNA CCHE1 silencing mediated the inhibition of ROCK1 expression

**Fig. 6:**
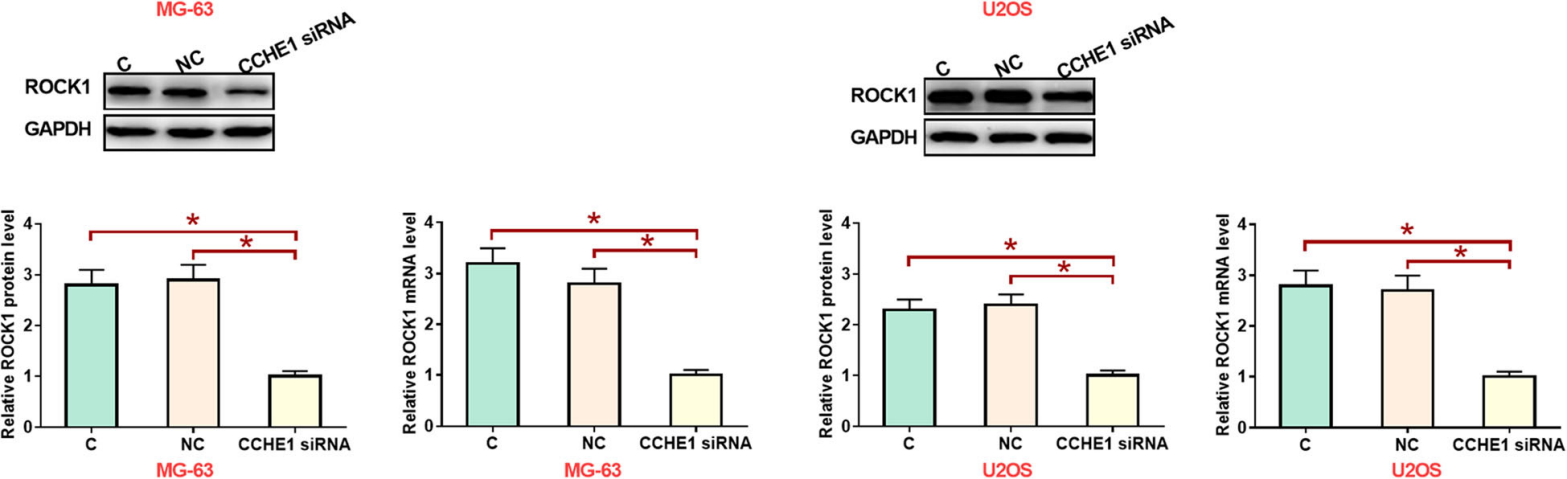
LncRNA CCHE1 silencing mediated the inhibition of ROCK1 expression (*, *p* < 0.05)

Western-blot results showed that, compared with control group (C) and negative control group (NC), lncRNA CCHE1 silencing led to significantly inhibited expression of ROCK1 in cells of both MG-63 and U2OS human osteosarcoma cell lines (Fig. 6, *p* < 0.05).

## Discussion

Postoperative recurrence of osteosarcoma is common [15]. The major finding of our study is that lncRNA CCHE1 may participate in distant recurrence but not in local recurrence of early stage osteosarcoma after surgical resection. We also provided experimental evidences that the actions of lncRNA CCHE1 are likely achieved through the interactions with ROCK1.

In spite of the development of early prediction approaches for cancer postoperative recurrence [16, 17], the accuracy is still low. In a recent study, Ying et al. reported multiple lncRNAs, such as RP1-261G23.7, RP11-69E11.4 and SATB2-AS1, can be used to predict recurrent osteosarcoma [18]. However, this study failed to distinguish local and distant recurrence. In our study we observed that lncRNA CCHE1 levels in plasma on the day of discharge were significantly higher in patients who developed distant recurrence than in patients with local recurrence and patients without recurrence. Therefore, detecting plasma lncRNA CCHE1 may provide guidance of follow-up chemotherapy or radiation therapy to prevent distant recurrence, which is a more malignant form compared with local recurrence.

As an oncogenic gene, ROCK1 is usually upregulated in the development of different types of cancers [7, 19]. Consistently, our study also observed significantly upregulated plasma ROCK1 in osteosarcoma patients than in healthy controls on the day of discharger. However, plasma ROCK1 was not differentially expressed in patients with different types of recurrence. Therefore, ROCK1 itself may not specifically participate in postoperative recurrence of osteosarcoma or at least have no predictive values for its recurrence.

ROCK1 participate in cancer biology not only through the interactions with proteins, but also by the cross-talk with non-coding RNAs, such as lncRNA [20, 21]. Interesting, our study observed positive correlation between plasma levels of ROCK1 and lncRNA CCHE1 on the day of discharge in patients who developed distant recurrence during follow-up. Our in vitro cell experiments data also revealed that lncRNA CCHE1 silencing can mediated the inhibited expression of ROCK1 in osteosarcoma cells, and lncRNA CCHE1 silencing may inhibit osteosarcoma cell migration and invasion through the downregulation of ROCK1. Therefore, inhibition of lncRNA CCHE1 expression may potentially be a therapeutic target for the treatment of osteosarcoma.

It is worth noting that we in this study only included a small number of patients and controls. To further analyze the reliability of the prediction of NSCLC distant recurrence by CCHE1, more clinical studies with bigger sample size are needed to further confirm the conclusion. Moreover, animal model experiments are also needed to confirm the functions of CCHE1.

## Conclusion

In conclusion, overexpression of lncRNA CCHE1 is involved in the distant recurrence of early stage osteosarcoma after surgical resection. The actions of lncRNA CCHE1 are at least partially achieved through the interactions with ROCK1.

## Data Availability

The analyzed data sets generated during the study are available from the corresponding author on reasonable request.

## Abbreviations

DR: Distant recurrence
lncRNA: Long non-coding RNA
LR: Local recurrence
NR: Non-recurrence
ROCK1: Rho-associated, coiled-coil-containing protein kinase 1
